# Gut microbiome and blood glucose control in type 1 diabetes: a systematic review

**DOI:** 10.3389/fendo.2023.1265696

**Published:** 2023-11-15

**Authors:** Jumana Abuqwider, Alessandra Corrado, Giuseppe Scidà, Roberta Lupoli, Giuseppina Costabile, Gianluigi Mauriello, Lutgarda Bozzetto

**Affiliations:** ^1^ Department of Clinical Medicine and Surgery, University of Naples Federico II, Naples, Italy; ^2^ Department of Molecular Medicine and Medical Biotechnology, University of Naples Federico II, Naples, Italy; ^3^ Department of Agricultural Sciences, University of Naples Federico II, Naples, Italy

**Keywords:** autoimmue diabetes, serum glucose level, time in range, glycated hemoglobin, 16S rRNA, shotgun sequencing

## Abstract

**Objective:**

The risk of developing micro- and macrovascular complications is higher for individuals with type 1 diabetes (T1D). Numerous studies have indicated variations in gut microbial composition between healthy individuals and those with T1D. These changes in the gut ecosystem may lead to inflammation, modifications in intestinal permeability, and alterations in metabolites. Such effects can collectively impact the metabolic regulation system, thereby influencing blood glucose control. This review aims to explore the relationship between the gut microbiome, inflammation, and blood glucose parameters in patients with T1D.

**Methods:**

Google Scholar, PubMed, and Web of Science were systematically searched from 2003 to 2023 using the following keywords: “gut microbiota,” “gut microbiome,” “bacteria,” “T1D,” “type 1 diabetes,” “autoimmune diabetes,” “glycemic control,” “glucose control,” “HbA1c,” “inflammation,” “inflammatory,” and “cytokine.” The examination has shown 18,680 articles with relevant keywords. After the exclusion of irrelevant articles, seven observational papers showed a distinct gut microbial signature in T1D patients.

**Results:**

This review shows that, in T1D patients, HbA1c level was negatively correlated with abundance of *Prevotella*, *Faecalibacterium*, and *Ruminococcaceae* and positively correlated with abundance of *Dorea formicigenerans*, *Bacteroidetes*, *Lactobacillales*, and *Bacteriodes*. Instead, *Bifidobacteria* was negatively correlated with fasting blood glucose. In addition, there was a positive correlation between *Clostridiaceae* and time in range. Furthermore, a positive correlation between inflammatory parameters and gut dysbiosis was revealed in T1D patients.

**Conclusion:**

We draw the conclusion that the gut microbiome profiles of T1D patients and healthy controls differ. Patients with T1D may experience leaky gut, bacterial translocation, inflammation, and poor glucose management due to microbiome dysbiosis. Direct manipulation of the gut microbiome in humans and its effects on gut permeability and glycemic control, however, have not been thoroughly investigated. Future research should therefore thoroughly examine other potential pathophysiological mechanisms in larger studies.

## Introduction

Type 1 diabetes (T1D) is an immune-mediated disease characterized by the progressive loss of insulin-producing β-cells in the islets of Langerhans within the pancreas (1). The shortage of insulin results in derangement of blood glucose homeostasis that may lead to potentially life-threatening acute and chronic complications (2). Triggers of the autoimmune damaging process are unclear. T1D incidence is rising globally, but there is a considerable country-to-country variance, with certain regions of the world experiencing a far greater prevalence than others (3). The reasons for this are unclear, but an interplay between genetic and environmental factors is strongly suspected (4). Despite the advances in T1D care, this disease continues to be associated with substantial medical, psychological, and financial burden. Additionally, hypoglycemia and hyperglycemia are persistent potentially life-threatening complications (5).

Recently, environmental variables such as gut microbiota, the complex microbial community that inhabits the human gut, have gained attention for their potential role in T1D pathogenesis. The human gut microbiome develops in the first few years of life, after which its makeup is similar to that of adults (6, 7). The maturation of the gut microbiome and immune system development are closely related processes (8). In 2011, According to research by Knip and colleagues on the relationship between the gut microbiome and T1D, children who had islet autoantibodies were more likely to have greater Bacteroidetes/Firmicutes ratios and lower Shannon diversity in their gut microbiome (9). Additional research revealed that children with a high risk of T1D have a considerable accumulation of the bacteria *Bacteroides dorei* and *Bacteroides vulgatus* (10) and associated with autoantibody positivity (11). Patients with T1D have lower concentrations of bacteria that produce lactate and short-chain fatty acids (SCFAs) (12). The reduced number of *Lactobacillus* and *Bifidobacterium* could also be detected at the onset of T1D (13).

Several cross-sectional case–control investigations have been performed to reveal the differences of gut microbiome between T1D patients and healthy control subjects. T1D children had larger *Bacteroidetes* and decreased abundances of the two predominant *Bifidobacterium* species when compared to healthy subjects (14). On the one hand, Mejía-León and Barca compared gut microbiome in patients with newly diagnosed T1D, patients with long-standing T1D duration, and healthy controls. The findings revealed that newly diagnosed T1D patients had higher levels of *Bacteroides*, while healthy controls had higher levels of *Prevotella* (15). On the other hand, only a few studies have investigated the role of the gut microbiome in long-standing T1D (16). The gut microbiome may play a crucial role in T1D pathogenesis by impacting intestinal permeability, and molecular mimicry, and modulating the innate and adaptive immune system (17). However, gut dysbiosis in T1D may play not only a pathogenetic role. Indeed, it could potentially influence blood glucose control in individuals who already have the disease. Research in type 2 diabetes patients or healthy subjects has revealed several proposed molecular mechanisms through which the gut microbiota can impact host glycemic control. These mechanisms include modulating incretin secretion, producing short-chain fatty acids, deconjugating bile acids, and regulating inflammation and function in adipose tissue (18). Nevertheless, there is limited knowledge about the specific relationship between gut dysbiosis and blood glucose control in type 1 diabetes and its underlying mechanisms. Hence, this review aims to explore the potential role of the gut microbiome in blood glucose control in T1D and its potential relationship with systemic inflammation and gut permeability.

## Methods

### Design of primary studies

A systematic review of observational studies was conducted to understand the correlation between gut microbiota composition and blood glucose control in T1D.

### Data source and search strategy

Every stage of the implementation and reporting of this systematic review were conducted according to the Preferred Reporting Items for Systematic Reviews and Meta-Analyses Guidelines (PRISMA 2009) (19).

Studies were found by searching the following data sources: Google Scholar (https://scholar.google.com), Web of Science (http://apps.webofknowledge.com), and PubMed (https://pubmed.ncbi.nlm.nih.gov), for observational studies on humans published in English language within the last 20 years. Manually scanning the lists of references from selected studies was undertaken to identify any additional relevant studies.

To achieve a high-sensitivity strategy, the following search words were utilized as both free-text and topic headings: “gut microbiota,” “gut microbiome,” “bacteria,” “T1D,” “type 1 diabetes,” “autoimmune diabetes,” “glycemic control,” “glucose control,” “HbA1c,” “inflammation,” “inflammatory,” and “cytokine.”

The criterion to explain the “observational study” was based on human experiments that may have been planned for an observation, and with purpose to determine the crosstalk between gut microbiota composition on fasting blood glucose, glycated hemoglobin (HbA1c), time in range, and other glycemic indices in T1D.

### Inclusion and exclusion criteria

Screening of abstracts and titles was the initial phase of the search, and the next phase consisted of testing full-text studies that met the following selection criteria: (1) human studies, (2) involvement of control group, and (3) exposure to T1D. The following exclusion criteria have been used: (1) not original paper, (2) lack of findings, and (3) animal studies. **Table 1** displays the PECOS (Population, Exposure, Control, Outcomes, Study design) guidelines.

**Table 1:** Criteria of PECOS standards for articles selection.

Standards	Inclusion	Exclusion
Population	Human, 0–70 years, with or without diabetes associated complication or other diseases.	Animal studies, pregnant, lactating, celiac, probiotic and prebiotic use, use medication affect gut microbiota like metformin, use antibiotic during the last 3 months, unusual eating habits (vegans).
Exposure	T1D more than 1 year	Prediabetic subjects with islet autoimmunity or not diagnosed with T1D or T1D <1 year
Control	Healthy subjects	
Outcome	Gut microbiota composition, glycemic indices (HbA_1c_, blood glucose level, time in range).	Poor procedure, unclear results, or other results.
Study design	Observational studies (cohort, case–control).	Interventional studies

### Data extraction

The following data were obtained for each article: author, publication year, sample size, participants age, study design, gut microbiota composition, gut microbiome assessment, and effect of gut microbiota composition on inflammation and blood glucose control in T1D.

### Quality appraisal

The Newcastle–Ottawa quality assessment scale (NOS) was used to test the quality and risk of bias in the studies (20, 21). A “star system” has been established in which a study is rated on three major angles: i) the selection of the study groups; ii) the comparability of the groups; iii) the ascertainment of either the exposure or outcome of interest for case–control or cohort studies, respectively. For each question in the Selection and Exposure categories, only one star may be given. For comparison, a maximum of two stars may be given. Based on their NOS scores, the scientific publications were given a “Good,” “Fair,” or “Poor” rating. High-quality studies were those with an overall rating of seven or higher. Studies scoring between 4 and 6 are considered adequate, while those scoring under 4 are considered inadequate (22).

## Results

### Selection of studies

**Figure 1:**
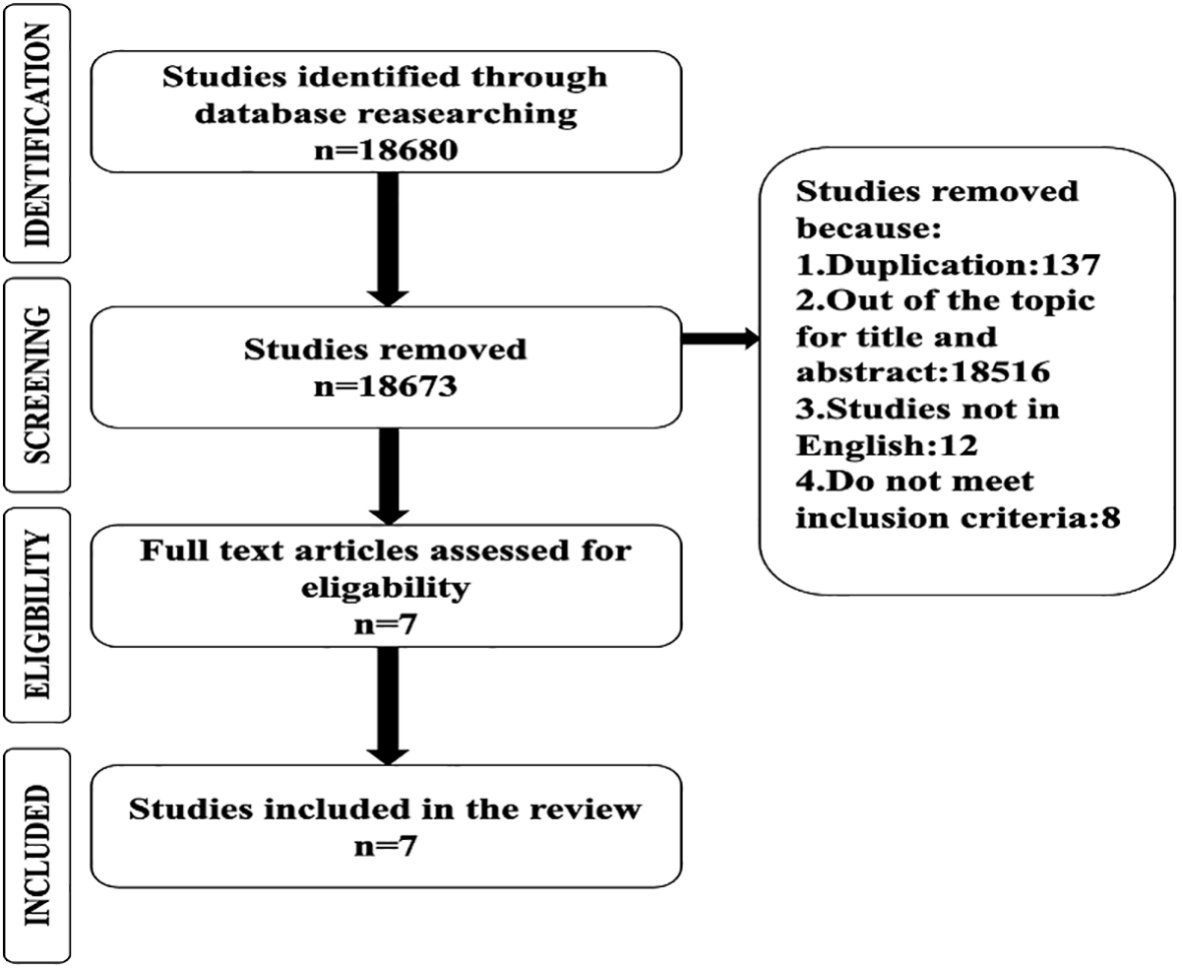
PRISMA flowchart showing the progression of articles through the various stages of the selection procedure.

Initially, a total of 18,680 articles were identified. Following the elimination of duplicates and articles deemed irrelevant to the topic or not meeting the inclusion criteria, 18,673 articles remained. Subsequently, seven studies were selected as the primary sources to test the hypothesis concerning the correlation between gut microbiota composition and blood glucose control in T1D (**Figure 1**). Upon assessment, the NOS scores for the included studies varied. Four articles were rated as good, receiving scores between 7 points, while two articles received fair ratings, ranging from 4 to 6 points. One article was considered poor. Notably, the risk of bias was lowest within the domain related to the definition of controls, and it was highest within the comparison domain, considering the subdivisions of the three domains.

### Main results of selected studies

In this section, we resume the main results of the papers selected in the review, and we organized them in three sub-sections. The first is focused on the gut microbiome composition of T1D patients and the differences with healthy subjects. The second is focused on the correlation between gut microbiota composition and blood glucose control. Finally, the third sub-section is dedicated to the markers of gut permeability and body inflammation that the authors investigated in T1D subjects.

#### Microbiome diversity in T1D patients and healthy subjects

In 2017, an observational study showed a different fecal microbiota composition between T1D patients (n=53) and healthy controls (n=50), all from the Amsterdam area in the Netherlands (23). Gut microbiota was assessed by V1–V2 16S rRNA gene sequencing. The study was conducted on 53 T1D patients and 50 healthy controls, all from the Amsterdam area in the Netherland. They reported no significant difference in species richness (alpha diversity) by comparing the Shannon’s diversity indices of T1D group and healthy group. Instead, the score plot from the Redundancy Analysis (beta diversity) visually showed a difference between T1D patients and healthy controls. Moreover, a Wilcoxon’s signed rank test was performed to evaluate the beta diversity per species, and the authors found that one genus, *Christensenella*, and one family, *Rhodospirillales*, significantly differentiate the fecal microbiota between T1D and controls. In particular, *Christensenella* appeared in 16% and 0% of T1D and controls, respectively, while *Rhodospirillales* appeared in 14% and 0% of T1D and controls, respectively. However, the low percentage of occurrence made this difference not significant when a false-discovery-rate corrected p-value (q-value) was calculated. Furthermore, the authors were able to locate multiple fecal species with high sensitivity that were predictive of belonging to either the T1D or the control group by using the elastic net technique.

A similar study was conducted 1 year later in Brazil but on a smaller number of subjects, i.e., 20 T1D patients and 28 healthy subjects (24). Gut microbiota composition was assessed by the sequencing of V3–V4 region of 16S rRNA gene. As in the previous study, these authors did not find a significant difference in alpha-diversity between the T1D group and healthy group, as well. A significant difference in beta-diversity was registered by the Unique Fraction (UniFrac) metric coupled with a standard multivariate statistical technique like the principal coordinate analysis (PCoA). Moreover, they compared the relative abundance of taxa in T1D and healthy groups and found a significant difference (*p*<0.05) in Betaproteobacteria class, Clostridiales order, *Ruminococcaceae* and *Lachnospiraceae* families, *Escherichia*/*Shigella*, *Bifidobacterium*, *Parabacteroides*, *Streptococcus, Bacteroides*, and *Clostridium* genera.

Huang et al. (25) analyzed 12 T1D patients and 10 healthy controls, all belonging to the Chinese Han ethnic group. They compared the gut microbiota composition by the sequencing of the V3–V4 region of the 16S rRNA gene. The alpha diversity of the microbiota, estimated by the total number of OTU and Chao, Shannon, and Simpson indices, was not significantly different comparing controls and T1D patients (25). PCoA of the beta diversity determined by UniFrac showed a separate clustering of healthy controls and T1D patients. Moreover, the authors found an inversion of the ratio between the two dominant phyla Firmicutes and Bacteroidetes, >1 in healthy group and <1 in T1D group. Finally, a linear discriminate analysis (LDA) effect size (LEfSe) identified distinct taxa between two groups. In T1D patients compared to healthy controls, LEfSe found 28 bacterial taxonomic clades with statistically significant changes (13 increased and 15 decreased). At the family level, *Ruminococcaceae*, *Veillonellaceae*, *Phascolarctobacterium*, and *Paenibacillaceae*, all belonging to the Firmicutes, were the most differentially abundant bacteria in healthy people, but *Porphyromonadaceae*, a family of the Bacteroidetes phylum, was overrepresented in T1D patients. Moreover, healthy subjects had a differential enrichment of the phylum Fusobacteria.

In line with the above-cited authors, Leiva-Gea et al. (26) reported no significant difference in alpha diversity (Chao index) but a significant difference in beta diversity (UniFrac PCoA) by comparing a group of 15 T1D affected children and 13 healthy control children, all of them from Spain (26). The 16S rRNA gene region V2–V3 sequences were analyzed for the comparison of gut microbiota composition. The ratio of phyla Firmicutes to Bacteroidetes was <1 in both groups, but T1D group showed a significant higher abundance of Bacteroidetes, 64.69% compared to 49.85% of the healthy group, while Firmicutes appeared significantly lower, 19.60% instead of 29.69% in healthy controls. In addition, the phylum Proteobacteria showed a significant lower abundance in T1D patients, 1.68% instead of 3.06%. The abundance of three families within both the Bacteroidetes (*Bacteroidaceae*, *Rikenellaceae*, and *Prevotellaceae*) and Firmicutes (*Ruminococcaceae*, *Veillonellaceae*, and *Streptococcaceae*) were significantly higher in T1D when compared to healthy control subjects. A significant strong lower abundance of *Lachnospiraceae*, a family of Firmicutes, was registered in T1D group, 22.1% compared to 42.0% of healthy group. A significant lower and higher abundance in T1D group was found for *Bifidobacteriaceae* and *Enterobacteriaceae* families, respectively. Finally, a significant increase in T1D patients was found at genus level for *Bacteriodes* and *Prevotella* (Bacteroidetes), *Ruminococcus*, *Blautia*, *Veillonella*, and *Streptococcus* (Firmicutes), and *Sutterella* and *Enterobacter* (Proteobacteria), while a significant decrease was found for *Lachnospira*, *Roseburia*, *Anaerostipes*, and *Faecalibacterium* (Firmicutes), and *Bifidobacterium* (Actinobacteria).

A group of 20 T1D patients and 11 healthy subjects from Tunisia were recruited in a study by Fasatouii et al. (27). They quantified six biomarkers (i.e., Firmicutes, Bacteroidetes, *Bifidobacterium*, *Akkermansia muciniphila*, and total bacteria) to compare gut microbiota of the T1D group with the healthy group (27). The authors carried out a quantitative PCR by using specific primers for each different biomarker to evaluate their abundance. They just observed a significant decrease in Firmicutes, *Faecalibacterium prausnitzii*, and *A. muciniphila* in T1D compared to controls.

A more structured work was performed by Shilo et al. (28), which analyzed gut microbiota composition of 74 adults with T1D, recruited in three medical centers in Middle East. The gut microbiota of T1D was compared with that of 296 healthy adults analyzed in a previous study (29). The authors extracted microbial DNA from stool samples and performed a shotgun analysis with 100 million reads per sample. Results of comparison between T1D and healthy group showed no significant difference in alpha diversity, species richness, and Firmicutes-to-Bacteroidetes ratio. Instead, the LDA showed a total of 17 and 15 bacterial taxa with significantly higher LDA score in T1D and healthy adults, respectively. However, the relevant result was that the prediction XGBoost-based model gave *Prevotella copri* as the most impactful microbial taxa toward the prediction of T1D, while *Ruminococcus* impacted the model toward the prediction of a healthy condition.

The last study that we included in this review was carried out by Van Heck et al. (30). These authors recruited 238 participants with T1D, all from outpatient diabetes clinic of the Radboud University Medical Center, the Netherlands, and aged over 18 years. The gut microbiota composition of this group was compared with that of 2,937 control subjects from the Lifelines Dutch Microbiome Project cohort. Shotgun sequencing was performed on approximately 8 Gb of 150 base pair paired-end reads per sample. Results showed no difference in alpha-diversity between T1D and controls, even though a small but significant increase in alpha-diversity of microbial biochemical pathway in the T1D group was found (30). Moreover, a total of 37 and 43 bacterial species in T1D patients were significantly increased and depleted, respectively, compared to the healthy cohort. Among the species involved in depletion, they found *Prevotella copri* and *Bifidobacterium longum*, while among those enriched, species belonging to *Ruminococcaceae* family were comprised.

#### Crosstalk between gut microbiota composition and blood glucose control in T1D

Not all authors found a correlation between gut microbiota changing and glycemic indices (**Table 2**). For instance, De Groot et al. (23) did not find any correlation between different fecal taxa and HbA1c. On the contrary, Higuchi et al. (24) discovered a negative connection in T1D patients between *Bifidobacterium* species and fasting blood glucose levels. Furthermore, associations between high HbA1C percentages and relative abundances of Bacteroidetes, Lactobacillales, and *Bacteroides dorei* were found.

**Table 2:** Correlation between blood glucose parameters and gut microbiome in T1D patients (**+** positive or **−** negative correlation).

Author	Year	Study design	Participants	Age (years)	Microbiome assessment	Blood glucose parameters vs. gut microbiome			
De Groot et al. (23)	2017	Case–control	53 T1D 53% M 50 HC 52% M	18–65	V1–V2	No correlation noticed			
Higuchi et al. (24)	2018	Retrospective cohort	20 T1D 14F/6M 28 HC 18F/10M	23±8.6 25±9.8	V3–V4	**−**	*Bifidobacterium*	➞	Fasting blood glucose
						**+**	Bacteroidetes	➞	HbA1c
						**−**	*Lactobacillales Bacteroides dori*	➞	HbA1c
Huang et al. (25)	2018	Case–control	12 T1D 5M/7F 10 HC 5M/5F	12–33	V3–V4	**−**	*Ruminococcaceae* *Faecalobacterium*	➞	HbA1c
Leiva-Gea et al. (26)	2018	Case–control	15 T1D 7M/8F 13 HC 7M/6F	<18	V3–V4	**+**	*Blautia* *Streptococcus*	➞	HbA1c
						**−**	Firmicutes/ Bacteroidetes	➞	HbA1c
Fassatoui et al. (27)	2019	Case–control	10 T1D 4M/6F 11 HC 3M/8F	20–67	Quantitative PCR	**−**	*Akkermansia*	➞	HbA1c
Shilo et al. (28)	2022	Retrospective cohort	74 T1D 28M/46F 296 HC 80M/216F	18–70	Shotgun	**−**	*Prevotellaceae*	➞	HbA1c
						**+**	*Enterobacteriales*	➞	Glucose average
						**+**	*Clostridiaceae*	➞	Time in range
Van Heck et al. (30)	2022	Retrospective cohort	238 T1D 129M/109F 2,937 HC 1,330M/1,090F	>18	Shotgun	**+**	*Dorea formicigenerans* *Ruminococcaceae*	➞	HbA1c
						**−**	*Fecalibacterium prausnitzii*	➞	HbA1c

In the study of Huang et al. (25), 10 healthy and 12 T1D Han Chinese subjects between the ages of 12 and 33 were recruited. Association analyses of clinical data and microbial community abundance demonstrated that abundances of *Ruminococcaceae* and *Faecalibacterium* were negatively correlated with HbA1c levels.

Leiva-Gea et al. (26) found many significant correlations between the relative abundance of some taxa and HbA1c in T1D children. For instance, the increase in *Blautia* and *Streptococcus* and decrease in the ratio of Firmicutes to Bacteroidetes were significantly correlated with increase in HbA1c level.

In 2019, another study (27) showed that the proportions of Firmicutes, *A. muciniphila*, and *F. prausnitzii*, and the ratio of Firmicutes to Bacteroidetes decreased in participants with T1D compared with those without diabetes. Furthermore, *A. muciniphila* was negatively correlated with glucose HbA1c level.

Shilo et al. (28) presented diverse findings in their study and employed multiple indices to assess the connection between clinical and microbial features and glycemic control measures, such as plasma fasting glucose and HbA1c levels (28). They observed significant associations between various bacterial taxa and glycemic indices. Specifically, they found a negative correlation between the relative abundance of certain unidentified *Prevotellaceae* species (e.g., SGB592 and SGB1340) and HbA1c levels, while a positive correlation was observed between *Enterobacterales* species (SGB2483) and average glucose levels. Additionally, species from the *Clostridiaceae* family (SGB1422) showed a positive correlation with time spent in the target glucose range.

Van Heck and her colleagues (30), in their research, identified significant associations between glycemic control, represented by HbA1c, and various microbial species. The study revealed a strong positive association with *D. formicigenerans* and strong negative associations with the genus *Fecalibacterium*, primarily dominated by *Fecalibacterium prausnitzii*. Moreover, HbA1c showed positive associations with bacteria from the family *Ruminococcaceae*.

HbA1c, or glycated hemoglobin, is a crucial indicator of long-term blood sugar control, and its concentration in the blood is an important glycemic index. Indeed, several large intervention trials showed that a decrease in HbA1c significantly reduce the risk of micro- and macrovascular complications over time.

#### Gut permeability and inflammatory parameters in T1D

De Groot et al. (23) quantified some biomarkers, related to gut permeability and inflammation, in plasma and fecal samples of 53 T1D patients and 50 healthy controls. Results did not show any difference in the markers of inflammation, like LPS-binding protein (LBP) and C-reactive protein (CRP) in plasma (23). The unique difference that they found between T1D and controls was in the level of acetate and butyrate in the plasma, which were significantly lower in T1D subjects. A further difference was found in the correlation between acetate and butyrate, which was positive in the T1D group and negative in healthy subjects. Additionally, the authors reported a positive correlation between HbA1c and plasma LBP (r=0.3) and a strong positive correlation between fecal IgA and fecal butyrate (r=0.65), both just in T1D patients (23). Interestingly, in terms of microbiota, they found that in T1D patients, *Christensenella* was negatively correlated with fecal acetate abundance, whereas *Subdoligranulum* was significantly correlated with plasma markers of endotoxemia LBP and inflammation CRP (23).

Higuchi et al. and his colleagues (24) used a cytometric bead array to evaluate the plasma concentrations of Interleukin-2 (IL-2), IL-4, IL-6, IL-10, IL-17A, interferon (IFN), and tumor necrosis factor (TNF) cytokines to determine the level of inflammation (24). The patient group’s plasma levels of IL-4, IL-6, and IL-10 were significantly higher than that of the control group. In order to assess correlations between the unbalance gastrointestinal bacteria and cytokines, the author analyzed the correlation between systemic levels of cytokines and reads percentages of bacterial groups present in stool samples from T1D patients. Interestingly, a significant correlation between the inflammatory cytokine IL-6 and *Ruminococcus* genus was identified. In addition, the TNF plasma levels negatively correlated with *Proteobacteria* and *Clostridiaceae* relative abundances. Lastly, the IFN-γ and TNF plasma concentrations correlated with relative abundances of *B. xylanisolvens* (24).

Leiva-Gea and her colleagues (26) investigated the possible increased gut permeability of T1D patients by quantifying the level of zonulin and LPS in the plasma. Moreover, they measured the inflammatory cytokines (IL-1β, IL-10, IL-6, TNF-α, and IL-13) plasma concentration. For all biomarkers, they found significant differences in the comparison between 15 T1D and 13 healthy children (26). Moreover, authors found many significant correlations between the relative abundance of some taxa and serum zonulin levels in T1D children. For instance, an increase in *Bacteroides* and *Veillonella* and a decrease in *Faecalibacterium* and *Roseburia* showed to be correlated with the rise in serum zonulin levels. However, the same authors assert that rather than a decrease in zonulin levels, the impaired gut permeability in T1D patients is more likely to be caused by *Veillonella*’s binding to colonic crypt cells. *Veillonella* pushes lactate to the luminal surface where it degrades tight connections. Moreover, significant correlations between serum levels of IL-1b, IL-6, TNF-a, IL-10, IL-13, and LPS and intestinal microbiota ecosystem were found in T1D. The increase in the relative abundance of *Bacteroides*, and *Veillonella*, and the decrease in *Bifidobacterium*, *Roseburia*, and *Faecalibacterium* in T1D were linked with serum IL-1b levels (26). Finally, serum IL-6 and TNF-a levels were correlated with an increase in the abundance of *Bacteroides* and a decrease in the abundance of *Roseburia* in T1D, whereas serum IL-10 and IL-13 levels were correlated with an increase in the abundance of *Streptococcus* and a decrease in the abundance of *Bifidobacterium*. Regarding the serum LPS, only the increase in the richness of *Veillonella* was related with its level in T1D.

**Figure 2:**
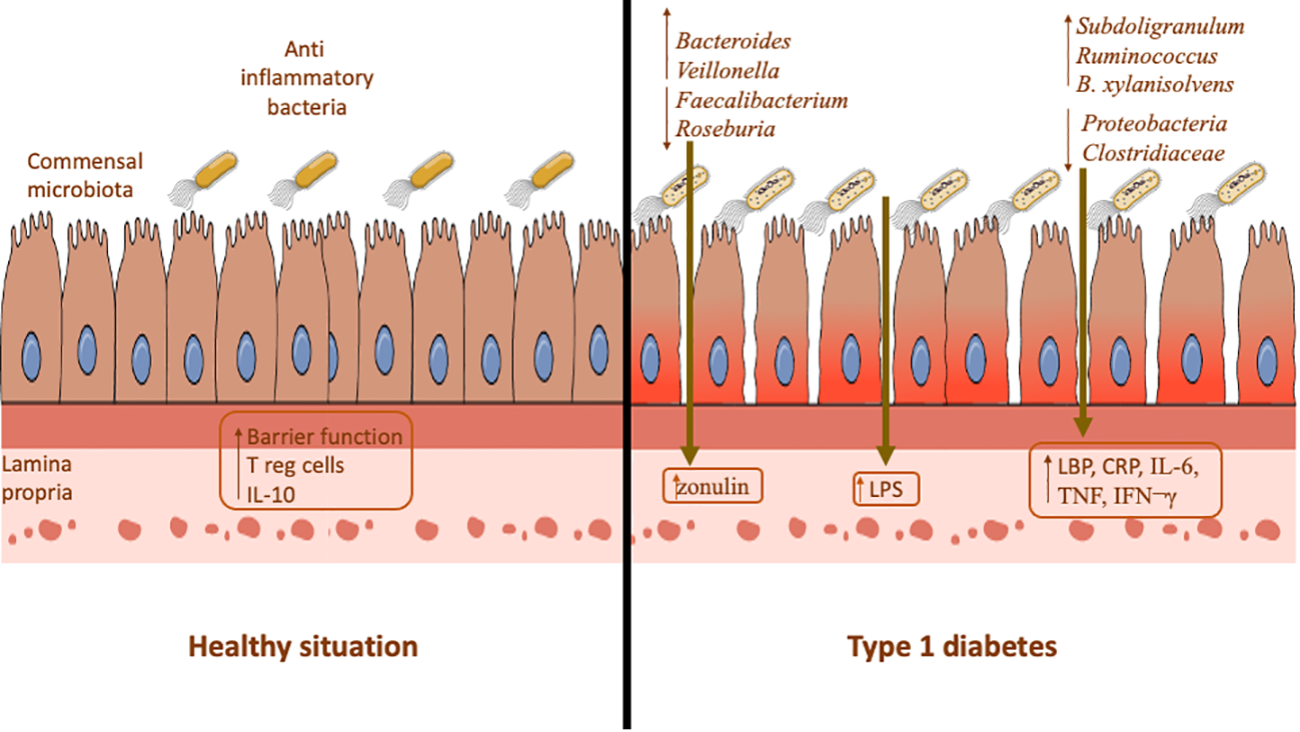
Comparison between healthy situation and type 1 diabetes in terms of gut microbiome, gut permeability, and inflammation parameters.

Collectively, all these findings confirm the idea that intestinal permeability and inflammation possibility are modified by gut microbiome, which in turn affect the T1D (**Figure 2**).

## Discussion

A rapidly growing number of studies have investigated the role of the gut microbiome in T1D pathogenesis in recent years (31–34), while only few studies have investigated the microbiome composition and its effect on the blood glucose control. As a matter of fact, all the studies selected in this review investigated the gut microbiota diversity and looked for correlations between changes in gut microbiota composition and some physiological parameters related to T1D. We must highlight that four (23–26) of the seven selected studies analyzed the gut microbiota composition by sequencing regions of 16S rRNA gene, one analyzed some microbial biomarkers representing the total gut microbiota (27), and just two (28, 30) of them performed a metagenome analysis by a shotgun sequencing strategy. As well currently recognized, the 16S rRNA gene partial sequencing can reveal differences between microbial niches just at family/genus level, making the differences at species level not so reliable, especially for very complex niches like the human gut. However, we have reported here the differences in the gut microbiota composition that these authors detected comparing T1D patients and healthy controls. Another important thing to underline is that the cohort size analyzed by these authors is very uneven, going from the minimum of 12 (23) to the maximum of 238 (30) T1D patients.

All authors (23–26, 28, 30), out of Fassatoui et al. (27), calculated and compared the alpha diversity of the gut microbiota composition in T1D and healthy groups. Microbiome alpha diversity is the first level of diversity measurement and is a key quantity in microbiome research. It gives an estimation of the taxa richness and evenness in a sample or group of samples. All the authors found not significant differences in alpha diversity between T1D and healthy groups. Nevertheless, this result is not in agreement with that of other similar studies, not included in this review, in which a significantly different alpha diversity was registered, both by 16S (35, 36) and shotgun (37) sequencing, comparing T1D and healthy subjects. In all the studies selected in this review, the beta diversity of gut microbiota was estimated. Generally, a linear discriminant analysis was performed to reveal the abundance diversity of specific taxa in the complex picture of the gut microbiome. By the combination of findings, also including other papers not selected in this review (12, 15, 23–28, 30, 36–38), the most reliable candidates as biomarkers of T1D dysbiosis are the genera *Bacteroides* (↑), *Bifidobacterium* (↓), *Dorea*, *Faecalibacterium* (↓), *Prevotella* (↓), *Roseburia*, and *Ruminococcus* (↓).

Just three out of the seven papers that we selected here investigated intestinal permeability and systemic inflammation in relation with microbiome composition and blood glucose control. Many studies reported in this review describe *Bacteroides* as one of the main genera leading to T1D-associated dysbiosis (12, 15). Therefore, *Bacteroides* could contribute to chronic inflammation by the impairment of the barrier function of the epithelial cell layer (39).

Even the growing use of continuous glucose monitoring (CGM) systems and use of different insulin regimen, the achievement of optimal glucose control and decrease in the risk of micro- and macrovascular complications in patients with T1D remains a great challenge.

An imbalanced gut microbiome promotes the proliferation of specific bacteria while hindering the growth of others. In this scenario, it has been documented that a certain bacterial species carried out anaerobic lactate fermentation, resulting in the generation of acetate, succinate, and propionate. As a consequence, this adversely impacts mucin synthesis, alters tight junctions, and disrupts intestinal permeability (40). As a matter of fact, *Akkermansia* leads to the increase in butyrate fecal concentration, which has beneficial effect on the gut integrity and glucose homeostasis (41). The oral supplementation of *Akkermansia muciniphila* significantly reduced the fasting blood glucose level, the OGTT area under the curve (AUC), the homeostatic model assessment for insulin resistance (HOMA-IR), and hepatic gene expression of G6Pase (an enzyme involved in glucose synthesis) (42). In our review, one study showed that the decline in *A. muciniphila* abundance leads to an increase in HbA1c and serum glucose level (27). Second, butyrate is one of the most significant consequences of microbial metabolisms and plays a crucial role in colonic T-reg induction, downregulation of pro-inflammatory macrophages, and integrity enhancement of gut barrier through increasing mucin production (43, 44). In this contest, *A. muciniphila* could be considered a beneficial bacterium and may improve glucose indices. Third, zonulin is a protein that can be assumed as an important indicator of mucosal integrity and gut permeability (26). By influencing zonulin, several bacterial groups can change mucosal integrity, increase *Bacteroides* and *Veillonella* genus, or decrease *Faecalibacterium* and *Roseburia* genus associates with increased serum zonulin levels in T1D patients (26).

Higuchi et al. (24) found a positive correlation between the abundance of *Bacteroides* and HbA1c, leading to the hypothesis that worse blood glucose control could be associated with an increase in serum zonulin and decrease in gut barrier integrity (24).

Certain authors have highlighted an altered Firmicutes-to-Bacteroidetes ratio in T1D patients, resulting in a shift in the overall gut microbiota composition and the relative metabolic equilibrium. Notably, the Bacteroidetes phylum’s anaerobic metabolism produces succinate and acetate, which can compromise epithelial tight junctions, reduce gut mucosal integrity, impede T-regulatory cell differentiation, and activate inflammatory pathways (45).

Actinobacteria, the next most abundant phylum after Firmicutes and Bacteroidetes, includes the genus *Bifidobacterium*. This taxon is known for producing butyrate with anti-inflammatory effects and enhancing the gut barrier through cytokine modulation (26). Moreover, *Bifidobacterium* stimulates T-regulatory cell development, leading to the suppression of immune responses by regulating IL-10 production (36). Interestingly, Higuchi et al. (24) found a negative correlation between *Bifidobacterium* and fasting blood glucose, suggesting that its anti-inflammatory properties could beneficially influence blood glucose control.

Another significant bacterium, *Faecalibacterium*, exerts anti-inflammatory effects and contributes to gut barrier function by modulating cytokine production and synthesizing butyrate (14). Although there is consistent evidence showing a strong negative correlation between *Faecalibacterium* and HbA1c, discrepancies emerged in the findings of Huang et al. (25) and Van Heck et al. (30) regarding the butyrate-producing family *Ruminococcaceae*. Huang et al. reported a negative correlation with HbA1c, while Van Heck et al. found a positive correlation (25, 30). As a result, further research is required to establish the correlation between *Ruminococcaceae* and blood glucose control.

Kostic et al. (46) reported that the abundance of *Blautia* was positively associated with glucose in the T1D infants, which was consistent with the results reported by Huang et al. (25) and Leiva-Gea et al. (26). In fact, these authors found that *Blautia* is positively correlated with HbA1c level.

**Figure 3:**
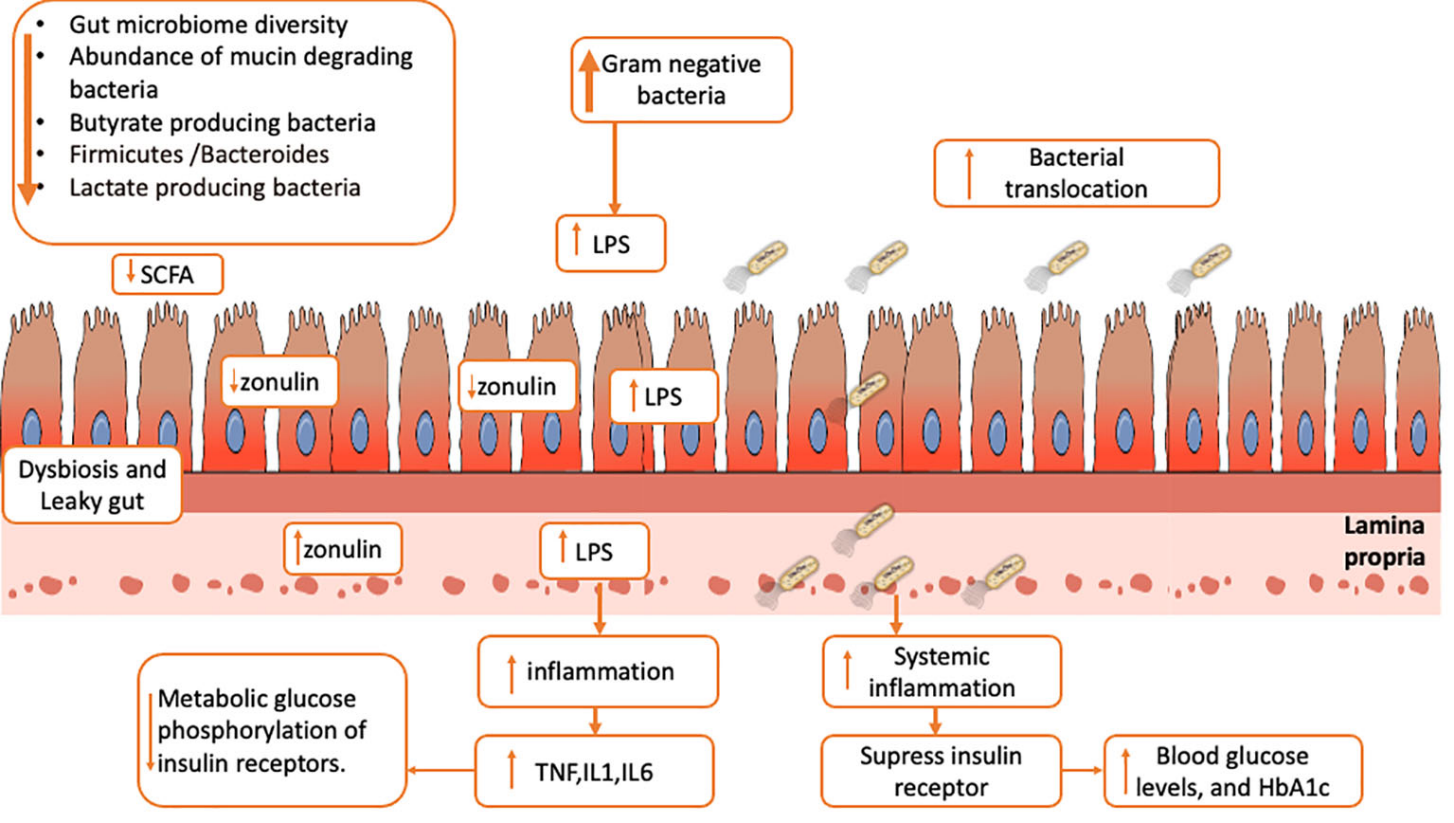
Possible link among gut dysbiosis, gut barrier permeability (reduction of zonulin and SCFA), LPS translocation across epithelial cells, systemic inflammation (increase of cytokines like TNF, IL1 and IL6), increase in metabolic glucose phosphorylation, and increase in blood glucose level.

Finally, our data indicate that 1) reduced diversity of gut microbiome, 2) reduced abundance of non-pathogenic mucin-degrading species, 3) reduced butyrate-producing members, 4) reduced Firmicutes-to-Bacteroides ratio, and 5) decreased relative abundance of lactate-producing bacteria may be all parameters able to identify a gut dysbiosis situation in T1D patients. Moreover, this picture may be strongly correlated with barrier permeability through a reduction in zonulin and SCFA production—especially butyrate—from Gram-positive members, which plays a crucial role in suppressing NF-κB signaling in the intestinal epithelial cells and stimulating T regulatory cells in the gut mucosa and enhancing the barrier *via* tight junction expression (44, 47) (**Figure 3**). Moreover, lactate-producing bacteria can perform several functions, including carbohydrate fermentation, acetate and lactate production, polyphenol and linoleic acid release, and antioxidant activity (48). They can also protect the gut from pathogenic bacteria by releasing bacteriocin, lowering luminal pH, and inhibiting epithelial adhesion (49). Based on this, we hypothesize that intestinal dysbiosis in T1D patients increases gut permeability and bacterial translocation, which can result in systemic inflammation, compromise insulin receptor function, and determine worse blood glucose control as shown by the association with high levels of HbA1c and blood glucose levels. Additionally, Gram-negative generated LPS binds to TLR4 and activates the inflammatory cascade, which causes NF-B to activate and release inflammatory mediators like TNF, IL-1, and IL-6, which affect glucose metabolism and block the phosphorylation of insulin receptors (50–52) (**Figure 3**).

## Conclusions

Finally, we conclude that there are different gut microbiome profiles between T1D patients and healthy controls. The microbiome dysbiosis in T1D patients could be involved in poor glucose control through leaky gut, bacterial translocation, and inflammation. However, direct manipulation of gut microbiome in human and its impact on gut permeability and glycemic control has not been well studied, so future studies should examine in detail and in larger studies other possible pathophysiological mechanisms to recognize specific pathways modulated by microbiota modulation and identify new potential therapeutic targets.

## Data availability statement

The original contributions presented in the study are included in the article/supplementary material. Further inquiries can be directed to the corresponding authors.

## Author contributions

JA: Conceptualization, Project administration, Writing – original draft. AC: Writing – original draft. GS: Writing – original draft. RL: Writing – original draft. GC: Writing – original draft. GM: Conceptualization, Funding acquisition, Supervision, Writing – review & editing. LB: Conceptualization, Project administration, Validation, Writing – review & editing.
